# Automated quantification of optokinetic responses based on head-movement

**DOI:** 10.1186/1471-2202-13-S1-P104

**Published:** 2012-07-16

**Authors:** Friedrich Kretschmer, Jutta Kretzberg

**Affiliations:** 1 Computational Neuroscience, Institute of Biology and Environmental Sciences, University of Oldenburg, D-26111 Oldenburg, Germany

## 

In computational ethology, the measurement of optokinetic responses (OKR) is an established method [1] to determine thresholds of the visual system in various animal species. Wide-field movements of the visual environment elicit the typical body, head and eye-movements of optokinetic responses. Experimentally, usually regular patterns, e.g. black and white stripes, are moved continuously. Variation of stimulus parameters like contrast, spatial frequency and movement velocity allows to determine visual thresholds. The measurement of eye-movements is the most sensitive method to quantify optokinetic responses, but typically requires the fixation of the head by invasive surgery. Hence the measurement of head-movements is often used alternatively to rapidly measure the behavior of many individuals. While an animal performs these experiments, a human observer decides for each stimulus presentation if a tracking reaction was observed or not [1]. Since responses of the animals typically are not recorded, off-line analysis and the evaluation of other response characteristics is not possible.

**Figure 1:**
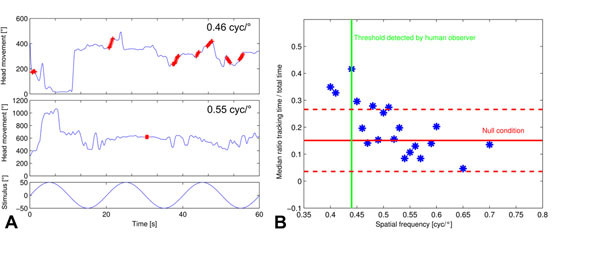
A: Head movements in response to sinusoidally moving stimuli of two different spatial frequencies. Red: Sequences, which were automatically identified as tracking behavior. B: Automatically identified tracking behavior at different spatial frequencies (blue: median, N=12) in comparison to random head movements in absence of a stimulus (red line: median, dashed: standard deviation) and to the threshold detected by a human observer (green).

We developed a method to automatically quantify OKR behavior based on the head movement in small vertebrates. For this purpose, we built a system consisting of a visual 360° panorama stimulation realized by four LCD monitors and a camera, positioned above the animal to record the head movements. A tracking algorithm retrieves the angle of the animal’s head. Here, we present a method for automated detection of tracking behavior based on the difference between the angular velocities of head and stimulus movement. Tracking performance is measured as the amount of time the animal performs head movements corresponding to the stimulus movement for more than 1s. For the optokinetic responses of mice we show that the tracking time decreases with increasing spatial frequency of a sinusoidal stimulus pattern (Fig 1). While a human observer was not able to detect tracking movements for spatial frequencies > 0.44 cyc/deg, the automated method revealed a certain amount of tracking behavior also at higher spatial frequencies. Thus, we were able to increase the sensitivity of the non-invasive measurement of optokinetic head movements into a sensitivity range that formerly required the measurement of eye movements.
